# Protective effect of microorganism biotransformation-produced resveratrol on the high fat diet-induced hyperlipidemia, hepatic steatosis and synaptic impairment in hamsters

**DOI:** 10.7150/ijms.59018

**Published:** 2022-09-11

**Authors:** Chun-Hung Su, Ho-Lin Wang, Min-Ling Tsai, Yi-Chun Lin, Jiuan-Miaw Liao, Cheng-Chieh Yen, Hung-Chih Ting, Ching-Han Yu

**Affiliations:** 1 Division of Cardiology, Department of internal medicine, Chung-Shan Medical University Hospital Taichung 40201.; 2 Institute of Medicine, School of Medicine, Chung Shan Medical University, Taichung 40201.; 3 Graduate Institute of Bio-industry Management, College of Agriculture and Nature Resources, National Chung Hsing University, Taichung 40227.; 4 Department of Pharmacy, Chung Shan Medical University Hospital, Taichung 40201.; 5 Department of Occupational Safety and Health, College of Health Care and Management, Chung Shan Medical University, Taichung 40201.; 6 Department of Physiology, School of Medicine, Chung Shan Medical University, Taichung 40201.; 7 Department of Early Childhood Educare, College of Health Sciences, TransWorld University, Douliu City, Yunlin County 64063.; 8 Department of Medical Research, Chung Shan Medical University Hospital, Taichung 40201, Taiwan.

**Keywords:** hepatic steatosis, hyperlipidemia, microorganism biotransformation-produced resveratrol, synaptic impairment.

## Abstract

**Background:** Resveratrol, a natural antioxidant polyphenol, has the functions of anti-inflammation, anti-cancer, liver protection and cardioprotection. Microorganism biotransformation-produced resveratrol (MBR) product shows higher purity than the natural source of resveratrol and costs less than the chemically synthesized resveratrol. The aim of the present study was to investigate the protective effects of MBR in hamsters treated with a high-fat diet (HFD).

**Methods:** MBR was obtained by the fermentative process of piceid. Hamsters were randomly divided into four groups: HFD plus oral administration of MBR 0 (C), 5 (L), 20 (M) or 50 mg/kg (H), respectively. After six-week of treatment, hamsters were sacrificed, and tissues were collected for further analysis.

**Results:** MBR at these three dosages did not influence the appetite or growth of the hamsters. Liver enzymes, blood glucose, total cholesterol, triglyceride, and liver weight were significantly reduced in the MBR groups than in the control group. Additionally, high-density lipoprotein-cholesterol (HDL-C) was also elevated in all MBR groups. On the other hand, serum low-density lipoprotein-cholesterol (LDL-C) was decreased in the MBR groups. Triglyceride (TG) in liver tissue and fatty liver level were lower in group H. Memory-associated proteins, phosphorylation of calmodulin-dependent protein kinase II (p-CaMK II) and synaptophysin (SYP), were increased in the brains of MBR groups.

**Conclusion:** The high yield- and short procedure-produced MBR has the potential to protect animals fed with HFD from hyperlipidemia, hepatic steatosis, hyperglycemia, and synaptic impairment, which might be beneficial for patients with these types of diseases.

## Introduction

Hyperlipidemia, such as higher levels of total cholesterol (TC), triglyceride (TG), and low-density lipoprotein cholesterol (LDL-C) and lower levels of high-density lipoprotein cholesterol (HDL-C), leads to multiple metabolic diseases. According to statistical data from the Ministry of Health and Welfare, Taiwan, there are five metabolic-related diseases on the list of the top 10 leading causes of death in 2021, including heart, cerebrovascular, and hypertensive diseases, diabetes mellitus, chronic-hepatic diseases and hepatic cirrhosis. In addition to Taiwan, diseases related to hyperlipidemia are also an important issue worldwide 1, 2. The mechanisms of how blood lipids result in these diseases are related to chronic inflammation and oxidative stress 3.

Cognitive impairment is one of the symptoms resulted from hyperlipidemia. Although brain cholesterol is involved in synapse development, dendrite differentiation and axonal elongation, people with familial hypercholesterolemia reveal higher incidence of memory deficits. On the other hand, patients with long-term statin treatment show better performance in episodic memory than control group 4, 5. Oxidized-LDL stimulates microvascular endothelial cells to secret inflammatory mediators which destroyed tight-junction structure of blood-brain barrier (BBB) 5. In addition, beta-amyloid peptides (Aβ) accumulation and the subsequent neuroinflammation, cerebral microhemorrhages, and cognitive decline may result from hypercholesterolemia 6.

Resveratrol is the non-flavonoid polyphenolic compound in grapes, peanuts, berries, and *Polygonum cuspidatum* (buffalo pea) 7, 8. Researchers are investigating its anti-inflammatory 9, antiapoptotic 10, antimicrobial 11, anticancer 12, antilipidemic 13, and osteogenic properties 14. The main function of resveratrol is to work as an antioxidant. The mechanism works to elevate the amount and activity of antioxidant enzymes and to reduce the lipoperoxidation 15. Accordingly, resveratrol prevents hepatic steatosis via decreasing reactive oxygen species (ROS) and regulating autophagy pathway in animal models 16, 17. Sirtuin-1 (SIRT1), a nicotinamide adenine dinucleotide (NAD^+^)-dependent deacetylase, is a key mediator of resveratrol for its antioxidant function. SIRT1 and its downstream transcription factor, forkhead box O1 (FoxO1), modulate autophagy induced by resveratrol 18. In addition, fasting blood glucose (FBG) and insulin resistance were significantly lower in patients taking resveratrol as a supplement 19. Furthermore, resveratrol protects brain function by lowering oxidative stress 20.

Natural sources of resveratrol may contain phytochemical impurities whereas chemically synthesized resveratrol may contain undesirable impurities. 21. Moreover, chemical synthesis requires expensive precursor and complicated multiple reactions and cleanup steps 22. In comparison, a fermentative process using yeasts such as *Aspergillus niger* or *Penicillium oxalium* to ferment precursor- or resveratrol-containing solutions provides higher yields than traditional methods. *P. cuspidatum* containing about 1~2% piceid (glycosylated resveratrol) is a applicable precursor for biotransformative producing of resveratrol 23. *Dekkera bruxellensis*, a yeast, presents highest β-glucosidase activity than other strains of microbes in our collaborator's previous study 24. Therefore, we used *D. bruxellensis* to ferment the root powder of *P. cuspidatum*. The fermentative product was called microorganism biotransformation-produced resveratrol (MBR). Syrian hamsters fed with high-fat diet (HFD) have been widely used as the animal model to examine the effects of dietary fibers, polyphenols, and other plant components on hyperlipidemia 25-27. The hypothesis of this study was that the MBR prepared from this study also revealed the bio-activities of pure resveratrol. Hence, three dosages of MBR were employed in this study to examine its effects on blood lipids, hepatic function, blood glucose, and synaptic function in hamsters fed with HFD.

## Materials and methods

### Materials

The yeast, *D. bruxellensis* was obtained from the Bioresource Collection and Research Center (BCRC 920084; Hsinchu, Taiwan). Yeasts were maintained in the yeast extract peptone dextrose medium (YPD broth) (BD, Sparks, MD, USA). Standard compounds of resveratrol and piceid were products of Sigma-Aldrich (St. Louis, MO, USA). Dried *P. cuspidatum* root was purchased from the local market (Taiwan) and ground into powder.

### Preparation of MBR

*D. bruxellensis* yeast was seeded at 5% in the YPD broth and shaken in a mechanical shaker at 150 rpm at 25°C for 3 days. Afterward, powdered *P. cuspidatum* root (2% w/v) and pre-incubated yeast (5% v/v) were added into the fermentation tank containing 20 mM acetic acid buffer solution (pH 6). The broth was incubated on the shaker at 150 rpm at 25°C with 1 vessel volume per minute (VVM) aeration for 0 to 64 hours. At each time point, the freeze-dried broth was weighed and extracted with 80% alcohol (1:20 w/v). After centrifugation, the liquid was filtered through a 0.45 µm membrane and was further concentrated by rotary evaporation (Sunway, Taipei, Taiwan). The concentrate was extracted with ethyl acetate twice. The ethyl acetate extract was again placed in the rotary evaporator. The concentrated powder was placed in the chemical hood until completely dry. The dry powder was then dissolved in 80% alcohol for the detection of resveratrol and piceid by high-performance liquid chromatography (HPLC). The column C18 (4.6 x 250 nm, 5 μm, Waters, Milford, MA, USA) at 40℃ was used with the eluting solvent of 1% acetic acid (A) and acetonitrile (B) at the flow rate of 1.5 ml/min. At the beginning, the proportion of eluting solvent was 95% A: 5% B. The proportion of solvent B was gradually increased to 55% after 25 min, and then continuously elevated to 100% from 25 to 31 minutes. From 31 to 33 minutes, 100% B was used. During the final 33 to 45 minutes, the proportion gradually turned back to 95% A: 5% B. The injected sample size was 20 μl. The wavelength of detected UV light was 307 nm. Resveratrol were detected at retention time of 18.3 minutes. 24.

### Animals and experimental groups

Six-week-old male Syrian hamsters, housed in a 12-h day/night schedule at 25° ± 2°C, were obtained from the National Laboratory Animal Center (Taipei, Taiwan). All animals could *ad libitum* access to the HFD (5TJN, TestDiet, St. Louis, MO, USA) and water. There are 40% of total calories from fat in this HFD. All animal experimental procedures were approved by the Institutional Animal Care and Use Committee (IACUC) at Chung Shan Medical University in Taiwan (No. 1613). Animals were divided randomly into four experimental groups of 10 animals each, including vehicle group (control [C], HFD only), low-dose group (L, HFD + 5 mg/kg MBR), medium-dose group (M, HFD + 20 mg/kg MBR) and high dose group (H, HFD + 50 mg/kg MBR). The amount of MBR fed was as suggested in a previous study 28. MBR was given to each hamster through gavage feeding every day for six weeks. This HFD-fed animal model which shows the increasing of body weight and hepatic steatosis in comparison to the standard chow diet-fed group has been established in our colleagues' previous study 29. Body weight, HFD and fluid intake were recorded once a week for six weeks. During the experiment, once the hamster showed distress, weakness or decreasing 15% of body weight, the animal was euthanized by pentobarbital sodium salt (intraperitoneal injection, 100 mg/kg) according to the American veterinary medical association (AVMA) guidelines for the euthanasia of animals: 2013 edition. At the end of this experiment, hamsters were fasted overnight and euthanized. The blood and organs were collected for further studies 30.

### Blood chemistry evaluation

Aspartate aminotransferase (AST), alanine aminotransferase (ALT), serum glucose, TC, TG, and HDL-C were determined by the biochemistry analyzer (Spotchem, Arkray, Kyoto, Japan). LDL-C was calculated by the following equation: LDL-C = TC - (HDL-C + TG/5) 31.

### Histological evaluation

At the end of this experiment, bilateral removal of epididymal white adipose tissue (EWAT) and the whole liver were performed. After weighting, part of liver tissue was homogenized to detect the hepatic TC and TG. Histopathological analysis was performed by Research Center for Animal Medicine (RCAM) of National Chung-Hsing University, Taichung, Taiwan. In brief, liver, heart, kidney, and pancreas were fixed in 10% formalin solution (pH 6.8-7.2) at 4ºC. Tissue sections were prepared by standard procedures and then stained with hematoxylin and eosin (H&E). The sections were analyzed by light microscopy. The criteria used for grading hepatic steatosis were described in the previous study 32.

### Immunoblotting analysis

Brain tissues were collected and divided into the cerebrum and cerebellum. Tissues were homogenized in a grinder in radioimmunoprecipitation assay (RIPA) buffer on ice for 30 minutes. After centrifugation at 10,000 ×g for 15 minutes of tissue lysate, the protein concentration of supernatant was determined by protein assay dye (Bio-Rad, Hercules, CA, USA). The proteins were separated by sodium dodecyl sulfate polyacrylamide gel electrophoresis (SDS-PAGE) and transferred to PVDF membranes (PerkinElmer, Waltham, MA, USA). After blocking, the membranes were incubated with primary antibodies, which including connexin 36 (Cx36) and phosphorylated Ca^2+^/calmodulin-dependent protein kinase (p-CaMKII, Santa Cruz Biotechnology, Santa Cruz, CA, USA), synaptophysin (SYP, Proteintech, Rosemont, IL, USA), and β-actin (Sigma), at 4°C for 16 h. After washing, the membranes were incubated with horseradish peroxidase-conjugated secondary antibody (Santa Cruz Biotechnology). The membranes were developed using enhanced chemiluminescence reagents (Perkin Elmer, Waltham, MA, USA).

### Statistical analysis

All data were given as the mean ± standard error (SE). Statistical analysis of results in Figure 2 was performed by two-way analysis of variance (ANOVA) using Duncan post hoc. In Figure 3~6 and Table 1, one-way ANOVA with post hoc Dunnett's test was used to analysis the results. A value of *P*<0.05 was considered statistically significant (Sigma-Stat 2.0, Jandel Scientific, San Rafael, CA, USA).

## Results

### Purity of resveratrol in MBR

**Figure 1:**
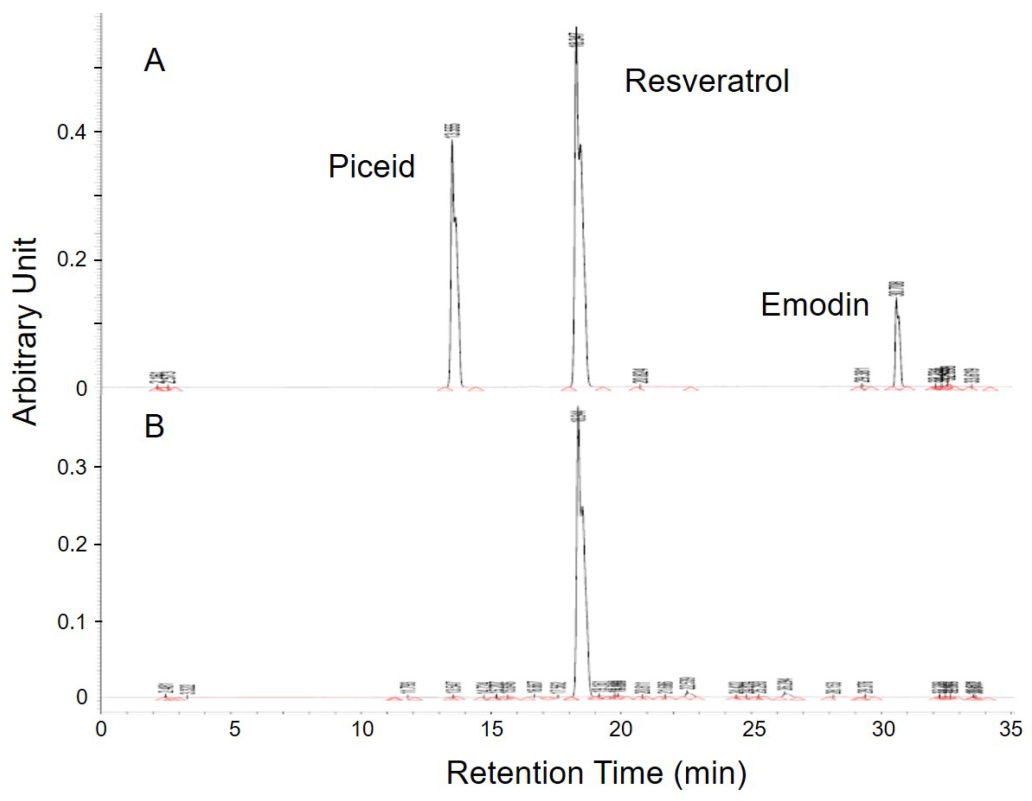
** HPLC analysis of MBR. (A)** Three standards were appeared at the retention time of 13.6, 18.3 and 30.7 minutes. **(B)** Among these three similar structures, resveratrol was significantly detected in the sample.

There were plenty of glycosides in the *P. cuspidatum* roots, which were an ideal base for the biotransformation-produced resveratrol. After screening for the activity of glucosidase in different strains, *D. bruxellensis* was selected to do the biotransformation of piceid to resveratrol. In the study of our collaborative team, the content of resveratrol reached the maximum level after 48 hours of incubation 24. The extraction of fermentative product was diluted and detected by HPLC to examine the concentration of piceid, resveratrol and emodin, which are the main phenolic compounds extracted from *P. cuspidatum* roots, according to the integration of area under curve in comparison with standards (Figure 1). The standard piceid, resveratrol and emodin appeared at the retention time of 13.6, 18.3 and 30.7 minutes. The average percentage of piceid, resveratrol and emodin in MBR sample was 0.3%, 67% and 0.03%, respectively. Therefore, the resveratrol concentration in MBR used in the animal study would be 3.35 (group L), 13.4 (group M) and 33.5 (group H) mg/kg/day.

### Lipid-lowering effect of MBR

**Figure 2:**
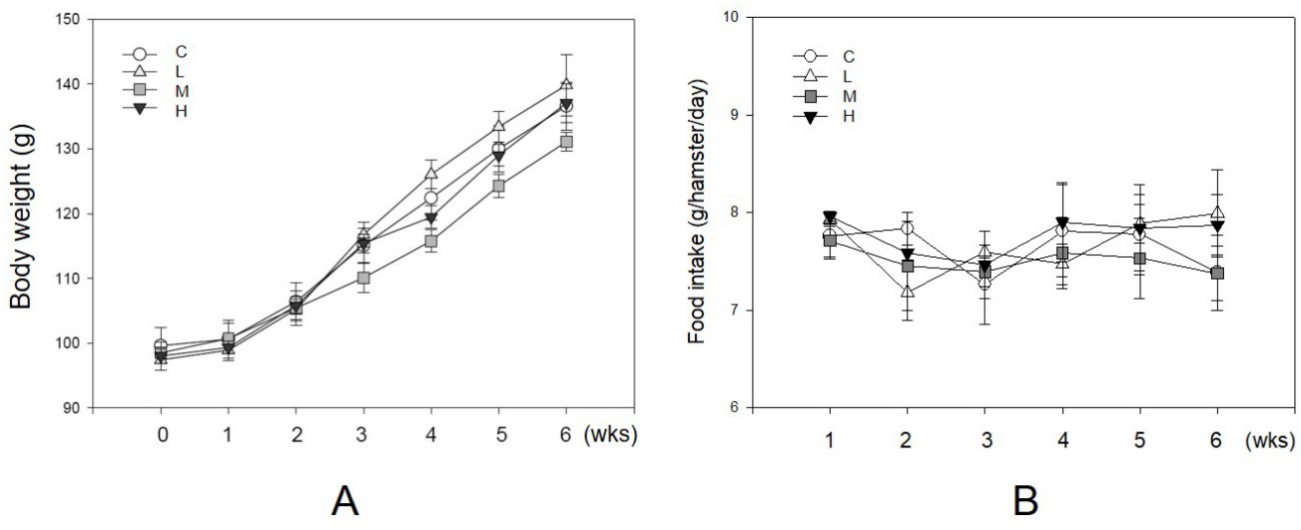
**Unaffecting of MBR on the body weight and food intake of hamsters during the experiment.** Hamsters were separated into four groups and fed with a HFD (control group, C), HFD + resveratrol 5 mg/kg (low dose group, L), HFD + resveratrol 20 mg/kg (medium dose group, M) and HFD + resveratrol 50 mg/kg (high dose group, H), respectively for 6 weeks. Body weight and food intake were recorded every week. **(A)** Body weight and **(B)** Food intake.

**Figure 3:**
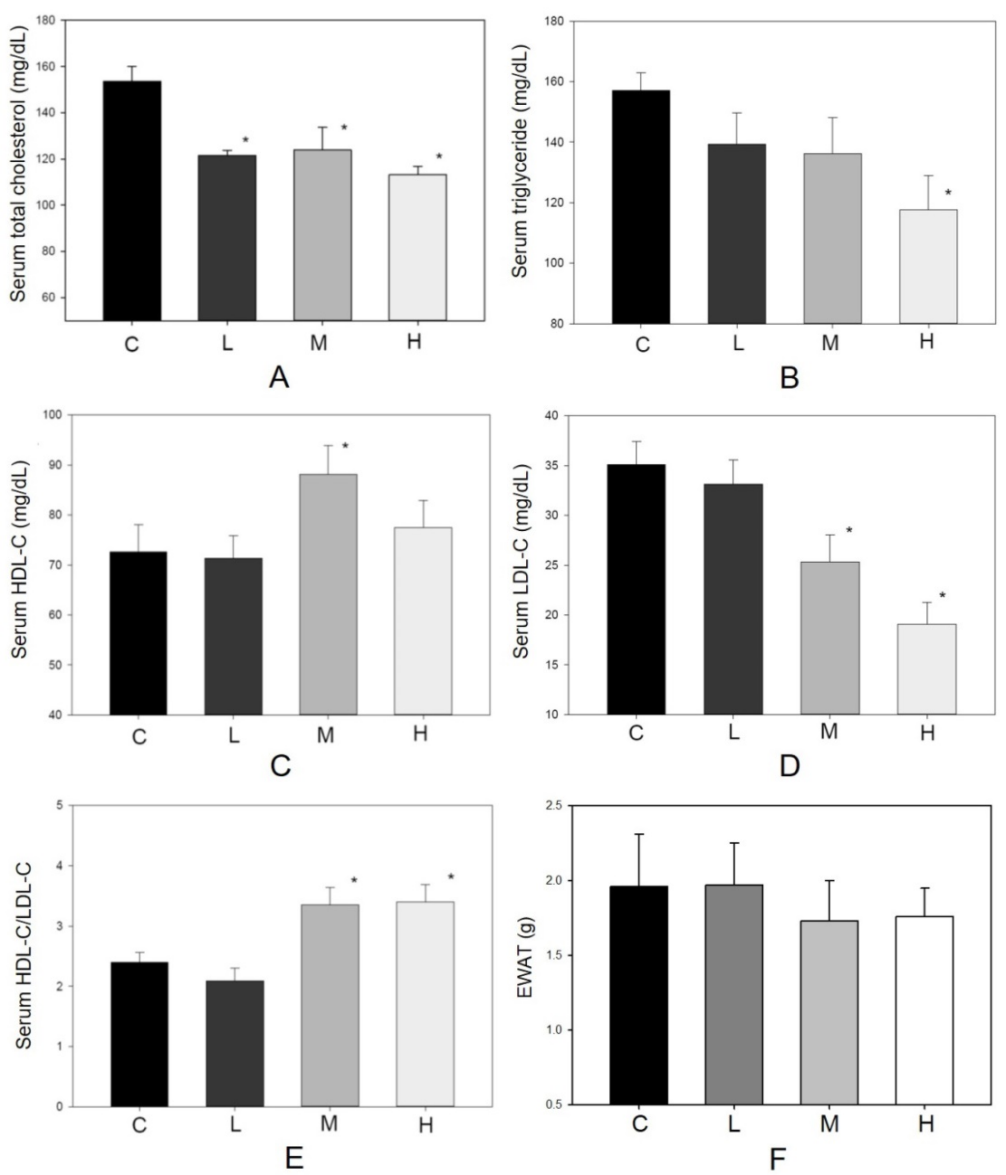
**Effect of MBR on lipid levels of hamsters fed with HFD.** Hamsters were fed with different diet for 6 weeks. After sacrificing, serum was collected and analyzed for** (A)** total cholesterol, **(B)** triglyceride, **(C)** HDL-C, **(D)** LDL-C and **(E)** HDL-C/LDL-C. **(F)** The weights of epididymal white adipose tissue (EWAT) were measured. Data are expressed as the mean ± standard error (SE). *P<0.05 vs. group C.

As an antioxidant, resveratrol shows a protective effect in lowering blood lipid concentrations 13, 17. During the experiment, no hamster showed significant adverse effects such as loss of body weight or reducing food intake (Figure 2). There were 10 hamsters in each group in the following analysis. Figure 3 shows the serum lipid levels including TC, TG, LDL-C, HDL-C, and HDL-C/LDL-C. In group L (5 mg/kg MBR), the lipid-lowering effect could only be found in the result of serum TC compared to the group C (control group)(Figure 3A). On the other hand, TC, LDL-C, and HDL-C/LDL-C in groups M and H significantly differed from that of group C (Figure 3A, D, and E). TG was significantly downregulated only in group H (Figure 3B). The weight of EWAT was used to represent the fat mass in the animal 33. The mean level of EWAT in groups M and H was 10% lower in comparison with group C. However, there was no significant difference between each group (Figure 3F).

### Anti-hepatic steatosis effect of MBR

**Figure 4:**
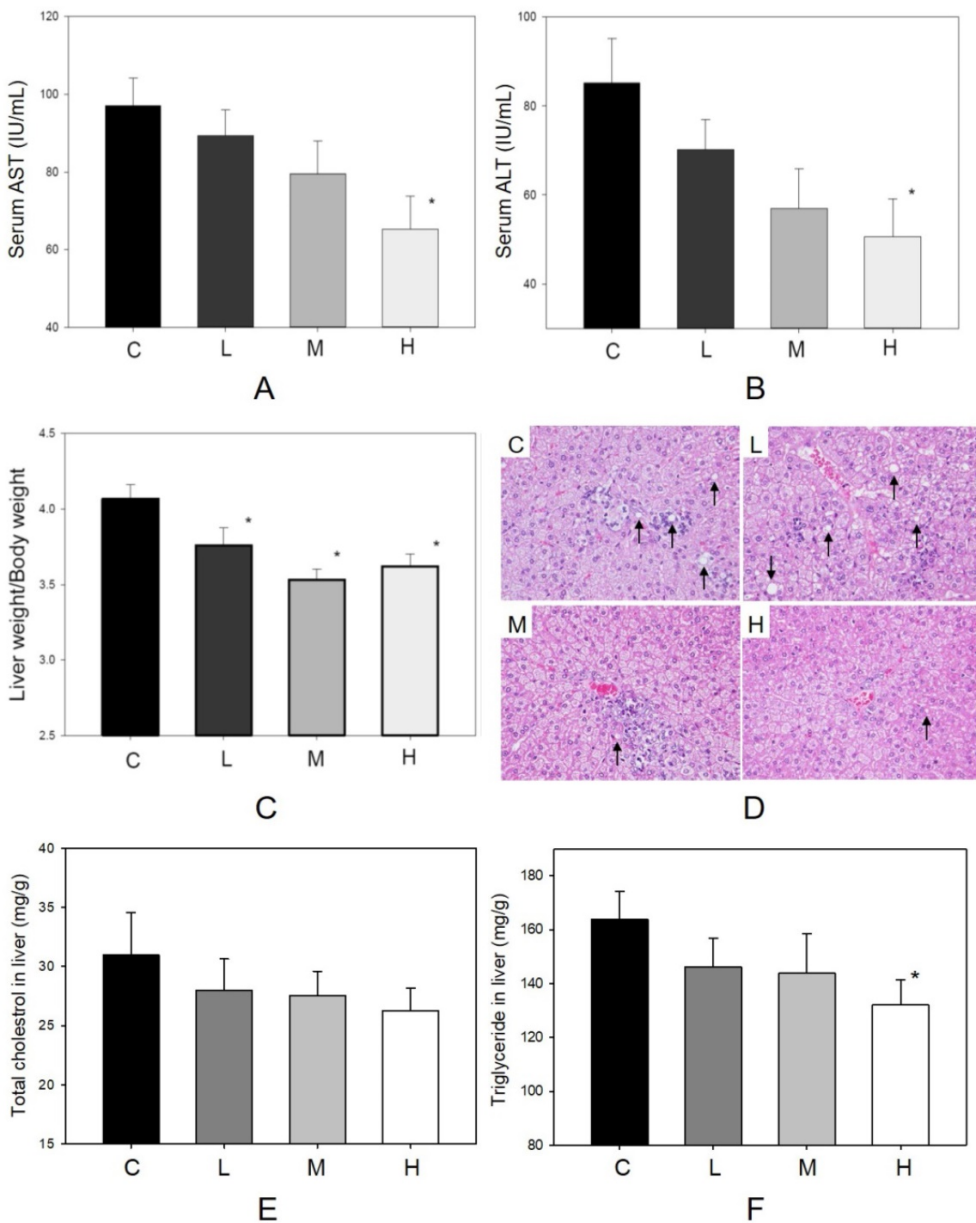
**Protective effect of MBR on liver function of hamsters fed with HFD.** Hamsters were separated into 4 groups and fed with HFD for 6 weeks. At the end of experiment, serum was gathered to examine the **(A)** AST and **(B)** ALT. Liver was collected to measure the **(C)** liver weight/body weight, to detect by **(D)** H&E stain, and to observe the **(E)** total cholesterol and **(F)** triglyceride in homogenized liver. Data are expressed as the mean ± SE. *P<0.05 vs. group C.

The factors influencing hepatic function and histopathological alteration of the liver were determined in four groups. In Figure 2A, body weight increased during the 6-week treatment period. The dosages of MBR treatment did not affect the appetite of hamsters (Figure 2B). In addition, administration of MBR resulted in a decrease in levels of serum AST and ALT compared to the control group, especially in group H (Figure 4A and B). MBR treatment also decreased the liver weight/body weight ratio in groups L, M and H in comparison to the group C (Figure 4C). Figure 4D and Table 1 show the results of the histopathological examination of the liver tissue. Fatty infiltration with micro- and macrovesicles in hepatocytes and mononuclear cell infiltration were found at different levels in the four groups. The severity was graded as 3.6 ± 0.7 in group C, 3.1 ± 0.6 in group L, 3.1 ± 0.6 in group M, and 2.7 ± 0.7 in group H. The significant lowering of severity could be found in group H (Table 1). TC and TG data of liver tissue were examined to represent the hepatic lipid accumulation (Figure 4E and F). The result showed that there were descending trends of both TC and TG in liver tissue in MBR-fed groups. The significant 19% reduction can be found in the result of TG in group H (Figure 4F).

### Blood glucose-lowering effect of MBR

**Figure 5:**
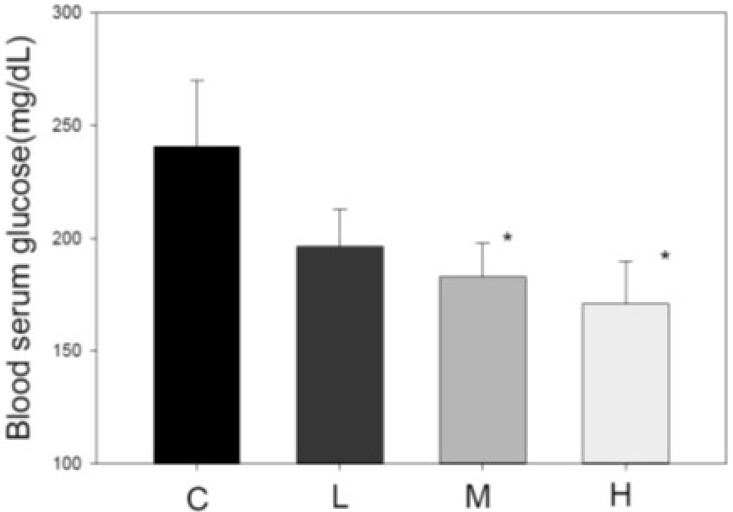
**Effect of MBR on blood glucose level in hamsters fed with HFD.** After feeding for 6 weeks, serum was collected to measure the glucose level. Data are expressed as the mean ± SE. *P<0.05 vs. group C.

Another index of metabolic syndrome is high blood glucose. HFD-fed animal model also revealed elevated blood glucose 34. After administration of MBR for 6 weeks, serum was collected for FBG examination. In HFD-treated group C, the glucose level was higher than that in the three MBR-treated groups. MBR supplementation in groups M and H significantly lowered glucose levels (Figure 5).

### Effect of MBR on synaptic function

**Figure 6:**
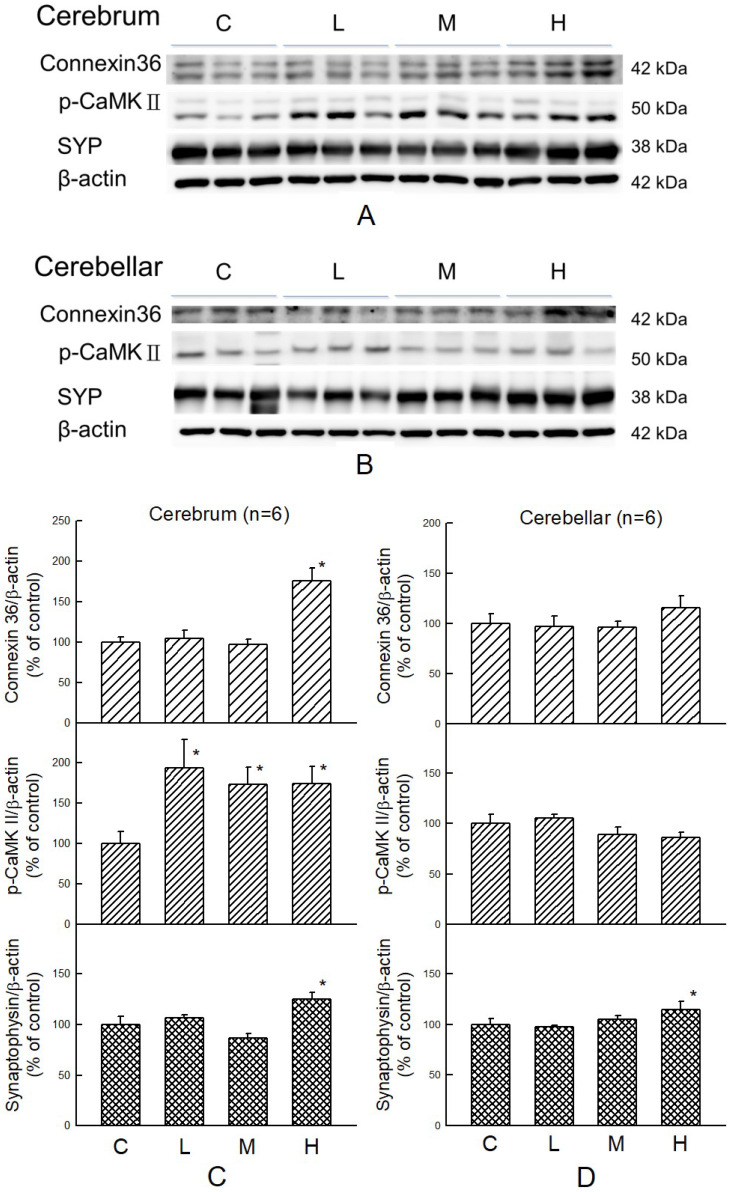
**Effect of MBR on the expressions of cognitive-associated proteins in the brain of hamsters fed with HFD.** After 6-week treatment, hamsters were sacrificed. **(A and C)** Cerebrum and **(B and D)** cerebellum were collected and analyzed by Western blot to detect the expressions of synaptic-related proteins, Cx36, p-CaMKII, and SYP. β-actin was used as a loading control. Data are expressed as the mean ± SE. *P<0.05 vs. group C. **(A and B)** Western blot; **(C and D)** Quantification data.

Cognitive-related proteins were analyzed in the cerebrum and cerebellum of HFD-treated hamsters with or without co-administration of MBR. In the cerebrum, which mainly controls the cognitive process, p-CaMKII, a kinase which is related to synaptic strength, was elevated for all three dosage groups of MBR. Cx36, a gap junction protein, and SYP, a presynaptic protein, were significantly increased only in group H for 74% and 26% in comparison with group C, respectively. On the other hand, in the cerebellum, which is less relevant to cognitive function, only SYP were significantly elevated for 18% in group H compared with group C (Figure 6).

## Discussion

Resveratrol usually shows as glycosylated form, also called piceid, in natural sources. Piceid could not present as many bioactivities as resveratrol. On the other hand, the product of bioengineered resveratrol needs complicated processes to remove the unexpected by-products 35. Therefore, to identify a microbe with efficient β-glucosidase and optimal fermentative formula may be a better way to obtain resveratrol in the industry. In the study of our collaborative team, the fermentative biotransformation of *P. cuspidatum* to MBR was optimum at about 48 hrs. Using 4-Nitrophenyl-β-D-glucopyranoside (pNPG) as a substrate to test the activity of β-glucosidase, *D. bruxellensis* showed high yield rate than other strains 24. During the analytic process of HPLC, the only minor percentage of piceid (0.3%) and emodin (0.03%) could be found in the sample (Figure 1). This result suggested that the effect of MBR was not from the piceid or emodin. The substances in the other 33% could not be detected by the HPLC method which we used in this study. During the fermentation process of yeast, saccharides were reduced to produce sugar alcohols 36. In comparison with sucrose, blood glucose and insulin levels are affecting less by sugar alcohols. This is the main mechanism of sugar alcohols to show the anti-diabetic and lipid-lowering effects 37. However, the sugar content in the diet used in this study was not replaced by MBR. Hence, protective effects revealed in the results were not from the by-product, sugar alcohols, in MBR. In addition, the lipid-lowering effect of pure compound of resveratrol and purified resveratrol has been reported in recent studies 38, 39. Therefore, we suggested that the results presented in this manuscript were mainly resulted from the resveratrol of MBR. Even though, the effects of by-product could not be totally ruled out since the direct result of by-product was not examined in this manuscript.

In the results, MBR ameliorated hyperlipidemia, hepatic steatosis, and hyperglycemia, and revealed neuroprotective effect in the HFD-treated hamsters. According to the references, a formula designed by pharmacologists to estimate dosages between species was using a conversion factor (km) based on the body weight/surface area of a species 40, 41. Therefore, the dose used in Species 1 is equal to (km Species 2/km Species 1) times of the dose in Species 2. In this study, MBR doses of 5, 20 and 50 mg/kg in hamster might be comparable to 0.42, 1.66 and 4.16 mg/kg in human, respectively. Although the results on hamster might not reflect directly on human, these amounts of MBR might be applicable in clinical usage.

Experimental studies indicate that HFD consumption by animals is a well-established model to study hepatic steatosis, insulin resistance 29, as well as impaired neurogenesis 42. Therefore, HFD-treated hamsters were used to examine the protective effect of MBR on the above disease-related factors. The decrease of TC and TG and increase of HDL-C/LDL-C were observed in the serum of HFD-treated hamsters supplemented with MBR (Figure 3A to E). However, EWAT weight which was usually used to represent the fat mass of the animal was not significantly reduced in MBR-fed groups (Figure 3F). According to the literature, the reduction of EWAT weight is not significant, but the size of adipocyte is 43. Therefore, MBR might also decrease the lipid accumulation in adipocyte in the current animal model. The downregulation of the lipids may result from decreasing fat accumulation and lipogenesis via modulation the transcription and activity of 5-hydroxy-3-methylglutaryl-coenzyme A (HMG-CoA) reductase by resveratrol 44, 45.

Metabolic syndrome is characterized not only by hyperlipidemia but also by hepatic steatosis. Dyslipidemia results in fatty infiltration and chronic inflammation of the liver. This effect may further extend to steatohepatitis 29. In group C, these indexes also appeared in the liver tissue sections (Table 1). In addition to reducing serum AST and ALT level, MBR treatment also ameliorated several hepatic lipid accumulated indexes, including hepatic fat droplets and hepatic TG level (Figure 4D and F). Resveratrol improves fatty liver disease and prevents hepatic steatosis via decreasing ROS and regulating the autophagy pathway 16, 46. Resveratrol and caloric restriction induced autophagy stimulated by the SIRT1-FoxO1 pathway has been identified as a critical protective mechanism of nonalcoholic fatty liver disease (NAFLD). Autophagy related genes are activated by the transcription complex of FoxO1 and other factors to remove of TG and lipid droplets in hepatic cells 16. Accordingly, MBR used in this study might also activate autophagy pathway in HFD-fed animal model to improve hepatic steatosis.

HFD induces obesity and dyslipidemia, which are risk factors associated with insulin resistance and hyperglycemia 19. The MBR produced in the present study displayed the ability to reduce hyperglycemia (Figure 5). It is reported that resveratrol reduces insulin resistance, fasting blood glucose, and inflammatory biomarkers 19. The mechanisms include increasing liver glycogen accumulation and elevating the expression, activity, and translocation of glucose transporter 4 (GLUT4)^47^. In addition, mitochondrial biogenesis elevates the glucose uptake in skeletal muscle. SIRT1 and its substrate peroxisome proliferator-activated receptor gamma coactivator 1-alpha (PGC-1α) stimulate the mitochondrial biogenesis which may be the glucose lowering pathway of resveratrol 48. Therefore, both GLUT4 and mitochondria in skeletal muscle might be the targets of MBR on lowering blood glucose.

Hyperlipidemia gives rise to oxidative stress by increasing lipid peroxidation. This lipotoxic effect of HFD treatment can damage human brain and cognitive functions 49. Additionally, lipid profile disorder also boosts the risk of vascular dementia, the second common cause of dementia after stroke 50. Resveratrol protects brain function by lowering oxidative stress and activates extracellular signal-regulated kinase (ERK) - cAMP response element-binding protein (CREB) pathway to preserve hippocampal CA1 neurons in ischemic reperfusion-treated rats 20, 51. CaMKII is necessary for synaptic function and behavioral memory formation 52. Cx36 is highly expressed in the nervous system and is an essential protein to form gap junctions, which facilitate the signal transport between neurons 53. SYP is the synaptic protein in the presynaptic element, and it can be used as a specific synaptic marker in the central nervous system. The amount of SYP may represent the quantity and quality of synapses 54. These three types of protein were upregulated in the MBR-treated groups, especially in group H. These results suggest that MBR might protect brain tissue from cognition impairment resulting from HFD via elevation of signal transmission between neurons.

## Conclusion

In conclusion, the MBR obtained by biotransformation, which is a high yield method, might have protective effects for hyperlipidemia, hepatic steatosis, hyperglycemia and synaptic impairment.

## Figures and Tables

**Table 1:** Diffuse of microvesicular fatty infiltration of liver in resveratrol-treated hamsters on a high-fat diet.

Organ	Histophathological Finding	Groups			
		C	L	M	H
Liver	Fatty infiltration with microvesicles, portal area, diffuse, slight to moderate/severe^1^	3.6±0.7^2^	3.1±0.6	3.1±0.6	2.7±0.7^*^

^1^Degree of lesions was graded from one to five depending on severity: 1 = minimal (< 1%); 2 = slight (1-25%); 3 = moderate (26-50%); 4 = moderate/severe (51-75%); 5 = severe/high (76-100%). ^2^The final numerical score was calculated by dividing the sum of the number per grade of affected hamsters by the total number of examined hamsters. ^*^ Statistically significant difference between control and treated groups at *P*<0.05.
